# Rapid Analysis of Sports Prohibited Substances in Black Market Pharmaceutical Products Using Atmospheric Solids Analysis Probe‐Mass Spectrometry

**DOI:** 10.1002/ansa.70088

**Published:** 2026-04-28

**Authors:** Alisha Henderson, Oliver Krug, Ashley Sage, David Douce, Scott J. Campbell, John Moncur, Mario Thevis, Liam M. Heaney

**Affiliations:** ^1^ School of Sport, Exercise and Health Sciences Loughborough University Loughborough UK; ^2^ Centre for Preventive Doping Research Institute of Biochemistry German Sport University Cologne Cologne Germany; ^3^ Waters Corporation Wilmslow UK; ^4^ SpectralWorks Limited Runcorn UK

**Keywords:** anti‐doping, deployable, drugs, mass spectrometry, screening

## Abstract

Misuse of performance‐enhancing substances in sport remains a persistent threat to the values of fair competition, with estimated doping prevalence far exceeding adverse analytical findings in routine testing. This highlights the need for improved surveillance tools capable of rapid and simple, on‐site screening methods. This proof‐of‐concept study evaluated atmospheric solids analysis probe‐mass spectrometry (ASAP‐MS) as a minimally complex, high‐throughput, and potentially deployable approach for detecting prohibited substances in black market products. Sixteen pharmaceutical products previously confirmed to contain prohibited substances were blind‐analysed using ASAP‐MS, alongside eleven third‐party batch‐tested sports supplements. Resultant multi‐channel spectra were interrogated manually for the presence of known precursor and product ions. In addition, a compound library was developed and applied using software‐driven matching. At least one prohibited substance was detected in all products without generating false positives in certified supplements. Manual spectral review identified only 68% of individual substances correctly. Initial issues with false positive detection of trenbolone in testosterone ester products using the software‐driven approach were iteratively optimised through alteration of match score thresholds. This achieved 96% correct substance identification and 100% detection for an adverse finding. Moreover, investigation of diagnostic precursor ions via heatmaps offered a complementary and additional reporting tool to reduce false negatives. This work demonstrated that ASAP‐MS, with tailored software‐driven analysis, provides reliable near‐real‐time qualitative screening of prohibited substances. More broadly, this supports the potential for portable, rapid mass spectrometry‐based screening platforms to strengthen forensic intelligence, accelerate anti‐doping investigations, and enhance athlete protection by complementing laboratory‐based confirmatory testing in sport.

## 1 Introduction

The intentional misuse of substances to aid performance within sport has been a concern within the industry for multiple decades, with data from elite sport estimating the prevalence of doping to be between 14% and 39% [1]. However, a disparity exists between the high level of assumed prevalence of doping in sport and the low detection rate of an adverse analytical finding (AAF) within testing programs (0.8% in 2023) [2, 3]. This highlights a critical need to continually improve anti‐doping surveillance, alongside iterative advancements in testing protocols, to combat the issue of intentional substance misuse. For athletes to achieve a goal of deliberate doping, they may source prohibited substances used for performance enhancement in sport from support personnel, friends, or external suppliers [4, 5]. Indeed, the World Anti‐Doping Agency (WADA) has discussed the black market as a prominent source for the procurement of products advertised to contain prohibited substances, with both amateur and elite athletes identified as clientele [6]. Furthermore, the issue of contamination and/or adulteration of products not marketed as medicinal in nature is also of concern, with an array of products purchasable via the black market being mislabelled [6]. In fact, the inadvertent consumption of prohibited substances in mislabelled products has frequently been cited by athletes as the cause for an AAF in doping control [7, 8, 9].

The presence of these aspects of deliberate and inadvertent doping places importance on the capacity of anti‐doping protocols to assess the presence/absence of prohibited substances in products purchased both legitimately and via unofficial routes. Critically, this can help accelerate the forensic questioning of suspect products seized from athletes/teams (i.e., deliberate use), as well as providing insight into the likelihood of a prohibited substance being present within nutritional aids assumed to be clean by athletes (i.e. inadvertent doping). For the latter, third‐party companies offer services to provide quality assurance tests on products, albeit that these processes are batch‐specific and at a cost to the manufacturers. Therefore, there is an ongoing interest in improving workflows to detect prohibited substances in products purchased through the black market, as well as those obtained through legitimate routes.

The use of hyphenated mass spectrometry (MS) approaches is the mainstay in sports anti‐doping protocols for both biological and non‐biological matrices [10]. However, these assays require trained personnel, alongside costly and bulky instrumentation, which are restricted to the laboratory environment. It is, therefore, of interest to improve the ability to qualitatively detect prohibited substances to offer screening approaches to complement current anti‐doping protocols. In particular, the capacity to analyse samples outside of the laboratory with a short turnaround time could specifically benefit the investigation of unknown or unlabelled products seized from athlete/teams. This, in turn, would accelerate forensic investigations through the rapid provision of the necessary information regarding the potential presence of a prohibited substance and subsequently flag the sample for confirmatory screening.

One analytical approach which has garnered increasing attention to address these forensic needs is the application of the atmospheric solids analysis probe (ASAP)‐MS [11, 12, 13]. ASAP‐MS is a well‐established ambient pressure‐based MS approach which offers rapid and direct analysis of samples with minimal sample preparation. When coupled with a single quadrupole mass analyser, ASAP‐MS provides a powerful analytical tool which requires only basic operational training, as well as having a small footprint, which facilitates its deployment outside of the analytical laboratory. This technique has already been showcased for its capability to identify the presence of prohibited substances in a non‐laboratory‐based environment, highlighting the potential for its use to translate into an anti‐doping context [14, 15, 16, 17, 18]. Importantly, although deployable and ambient MS platforms have been investigated for forensic and illicit drug identification, the application of these workflows within a sports anti‐doping context remains limited. In particular, the integration of ASAP‐MS with substance‐specific compound libraries and optimised search algorithms would enable the rapid screening of products suspected to contain WADA‐prohibited substances. This approach would positively expand the options available within sports anti‐doping investigations.

In this proof‐of‐concept study, products with performance‐enhancing capability sourced via black market routes known to be available to athletes were analysed by ASAP‐MS. This approach was developed by combining a purpose‐built, multi‐channel spectral library with an optimised software‐driven compound matching workflow. Overall, this aimed to assess the applicability of a high‐throughput, minimal sample preparation MS‐based approach to confidently identify the presence of known prohibited substances, and to understand the potential utility of ASAP‐MS to act as a technique to complement and add to current anti‐doping workflows. Therefore, this work provides the first structured assessment of an integrated, deployable ASAP‐MS screening workflow specifically tailored to product surveillance within sports anti‐doping.

## 2 Material and Methods

### 2.1 Materials

Methanol (MeOH) was purchased from Fisher Scientific (Loughborough, UK). Sixteen performance‐enhancing products were purchased via the black market and previously analysed and confirmed for advertised content by liquid chromatography‐tandem MS (LC‐MS/MS) at the Centre for Preventive Doping Research, German Sport University Cologne (Cologne, Germany) [19]. These products consisted of seven oily solutions, eight solid tablets, and one suspended solution. All products were confirmed to contain at least one substance prohibited by WADA (Table 1). Chemical standards used for the *Compound Library* included testosterone propionate (Product Code: T1875, purity 100%), testosterone isocaproate (T026000, European Pharmacopoeia (EP) Reference Standard), trenbolone (T3925, analytical standard, purity 100%), clenbuterol hydrochloride (C5423, purity 98.4%, racemic mixture), clomiphene citrate salt (C6272, analytical standard, 60% trans isomer), and ostarine (Advanced ChemBlocks Inc, ADV428292944, purity 98%, S isomer) purchased from Merck (Gillingham, UK), with testosterone 17‐phenylpropionate (TRC‐T155150, purity 98%), testosterone decanoate (DRE‐C17322535, purity 98%), and testosterone undecanoate (LPM‐TES‐1253, purity 99.9%) purchased from LGC Standards (Guildford, UK). Nutritional supplements certified as clean through a UK‐based testing and certification programme (Informed Sport, LGC, Fordham, UK) were obtained from volunteers within the research facility. The nutritional supplement products consisted of one gel capsule, three tablet capsules, three solid tablets, three powdered solids, and one liquid solution. Closed soda glass capillary tubes (Sanco G119/32) were obtained from Fisher Scientific (100 mm length, 1.8 mm outer diameter).

**TABLE 1:** Details of the pharmaceutical products purchased via the black market, including product formulation, presence of prohibited substances, and concentration/mass of prohibited substance. The S codes within brackets relate to the substances category within the World Anti‐Doping Agency *Prohibited List* (S1 – Anabolic agents, S4 – Hormone and metabolic modulators).

Sample ID	Product formulation	Prohibited substance(s) present	Concentration
1	Oily solution	Testosterone undecanoate (S1)	40 mg/mL
2	Oily solution	Testosterone undecanoate (S1)	40 mg/mL
3	Oily solution	Testosterone undecanoate (S1)	40 mg/mL
4	Suspension	Trenbolone (S1)	50 mg
5	Oily solution	Testosterone propionate (S1)	30 mg/mL
		Testosterone phenylpropionate (S1)	60 mg/mL
		Testosterone isocaproate (S1)	60 mg/mL
		Testosterone decanoate (S1)	100 mg/mL
6	Oily solution	Testosterone propionate (S1)	30 mg/mL
		Testosterone phenylpropionate (S1)	60 mg/mL
		Testosterone isocaproate (S1)	60 mg/mL
		Testosterone decanoate (S1)	100 mg/mL
7	Oily solution	Testosterone propionate (S1)	30 mg/mL
		Testosterone phenylpropionate (S1)	60 mg/mL
		Testosterone isocaproate (S1)	60 mg/mL
		Testosterone decanoate (S1)	100 mg/mL
8	Oily solution	Ostarine (S1)	50 mg/mL
9	Tablet	Clenbuterol (S1)	40 µg
10	Tablet	Clenbuterol (S1)	20 µg
11	Tablet	Clenbuterol (S1)	40 µg
12	Tablet	Clenbuterol (S1)	40 µg
13	Tablet	Clomiphene (S4)	50 mg
14	Tablet	Clomiphene (S4)	50 mg
15	Tablet	Clomiphene (S4)	50 mg
16	Tablet	Clomiphene (S4)	50 mg

### 2.2 ASAP‐MS Parameters

All analyses were performed using a RADIAN ASAP Direct Mass Detector (Waters Corporation, Milford, MA, USA) consisting of a dedicated ASAP probe loading holder for insertion into an atmospheric pressure chemical ionisation (APCI) ion source coupled with a single quadrupole mass analyser. Ionisation via APCI was performed in positive (+) ion mode with the system operated in performance mode. The source temperature was set to 150°C, the corona current at 3 µA, and the N_2_ gas heater temperature at 450°C. Each scan was completed across a mass range of *m/z* 50–600 in continuum mode at a sampling frequency of 5 Hz. In order to induce in‐source fragmentation, all analyses applied a multi‐function scan mode consisting of four data channels of rising cone voltages set to 15, 25, 35 and 50 V. Data analyses were performed using MassLynx 4.2 (Waters Corporation), MSP Librarian (v2.0), and AnalyzerPro XD (v1.16, SpectralWorks, Runcorn, UK). The total analysis time was ∼5 min, with three separate insertions of the sample across each analytical run.

### 2.3 Compound Library Development

Each chemical standard was analysed as a solution of 100 µg/mL in MeOH by dipping the glass capillary tube into the solution before transfer to the ASAP‐MS system. The acquired spectra across each scan channel (i.e. differing cone voltages) were used to build a spectral library. The acquired continuum data were binned to the nearest Dalton using the mass measure function in MassLynx and uploaded to MSP Librarian to build a four‐channel mass spectral profile for each prohibited substance. Following the addition of all chemical standard spectra, the MSP Library file was opened in the AnalyzerPro XD software and saved in its own target library format for use during processing. Data files were imported into AnalyzerPro XD using the *Direct MS* function, with default search settings using the top four masses in each channel set at a mass window of 0.3 amu, a match score threshold of 80%, and a 70:30 weighting for the forward and reverse library score matching with a threshold of 750 and 500 for the forward and reverse match score, respectively. Search weightings across the four cone voltage data channels (used to calculate the overall match score confidence) were set at 10%, 15%, 45%, and 30% for 15, 25, 35, and 50 V cone voltages, respectively. This was performed to provide an improved capacity to differentiate compounds based on their in‐source fragmentation products, reducing the impact of common precursor ions at lower cone voltages. Chemical information of the analysed prohibited substances, including known precursors and product ions, is provided in Table S1.

### 2.4 Sample Preparation and Analysis

All liquid/oily products were diluted 1:200 (v/v) in MeOH to prevent source contamination and detector saturation, with solid samples analysed in their native powdered form or first ground into a powder using a pill crusher. Glass capillary tubes were preconditioned to remove any potential contamination using the default instrument workflow by inserting the probe into the ion source under a stream of N_2_ at 600°C. After allowing time to cool (∼30 s), the glass capillary tube was dipped directly into the sample, placed onto the sample loading holder, and immediately inserted into the ion source. Each insertion of a sample remained within the ion source housing until manual monitoring of the total ion chromatogram showed total or near complete desorption of analytes by return of the signal intensity to the baseline, pre‐insertion intensity. All samples were initially analysed in a blinded manner, with a priori knowledge of the list of substances present but without knowledge of the contents of each sample.

### 2.5 Manual Interrogation of Spectra

To understand the ability to immediately assess the sample spectra and assign confidence to identify the presence of a prohibited substance(s), each sample was manually interrogated by a trained ASAP‐MS user. Mass spectra were visually investigated for the presence of known precursors and product ions for each potential prohibited substance. The known diagnostic ions used for compound identification were obtained from the previously performed LC‐MS/MS analyses and are reported in Table S1. A substance was considered present when there was clear evidence of the presence of the [M+H]^+^ precursor ion in the 15 V cone voltage data channel, and at least two known product ions present above baseline noise levels across the remaining data channels.

### 2.6 Compound Library Matching

In addition to manual searching of spectra by a trained user, a software‐driven library searching approach was applied. For this, MassLynx analysis files (.raw format) containing three repeat analyses of the pharmaceutical products or nutritional supplements were imported into AnalyzerPro XD. Each ASAP‐MS acquisition file was imported individually and was split into three separate analysis files using the *data split* function, which automatically isolated each individual peak within the total ion chromatogram (TIC). This was performed using the *continuous flow ionisation gas mode* and Gaussian smoothing set to three. A sequence file containing each isolated peak was generated and processed using the *Direct MS* function with automatic *m/z* detection. The method was configured to produce an average spectrum across 22 scans (including 9 scans prior to the apex, the apex scan, and 12 scans following the apex). No background subtraction was applied. A height threshold of 0.5% and a *m/z* window of 0.5 were used. The sequence file(s) were linked to the developed compound library, and library searching was enabled to compare spectral profiles within each sample against prospective prohibited substances. The library matching workflow outputted an individual result for each split peak, detailing the matching of any library entries alongside a search confidence value. The threshold for a positive identification of a library entry was set to 80%. In addition to library matching, heatmaps were produced to target precursor ions to provide a secondary visualisation for the likelihood of the presence of a known substance. Following analysis of the pharmaceutical products and nutritional supplements (and unblinding of sample contents), alterations related to library searching were performed to optimise the detection of the known prohibited substances.

### 2.7 Data Analyses

The number of successful substance identifications using both a manual and software‐driven approach was calculated and evaluated. Values for the total number of correct substance identifications (i.e. positive identification of a substance wherever present), correct sample identifications (i.e. positive identification of all substances present within a sample), and correct adverse finding (AF) (i.e. positive identification of at least one substance present in a sample) were collated. Certified clean nutritional supplements were classified as incorrect if any substance was considered present (i.e. a false positive). Substance identification data were subsequently assessed for diagnostic performance through the calculation of diagnostic sensitivity and specificity, as well as the positive predictive value (PPV) and negative predictive value (NPV). Concordance statistics were calculated to show a measurement of discrimination between the predicted and actual test outcome using IBM SPSS Statistics (v.29, IBM, Armonk, NY, USA).

## 3 Results

**FIGURE 1:**
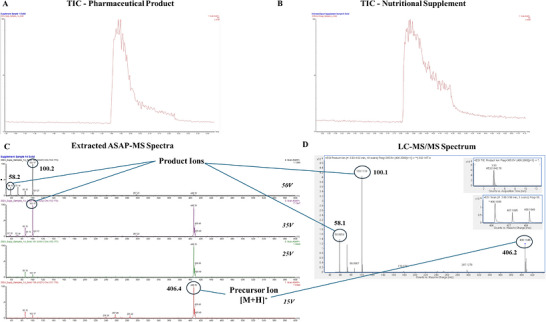
Total ion chromatogram (TIC) from the analysis of (A) a black market pharmaceutical product containing clomiphene and (B) a certified clean nutritional supplement, alongside (C) an example of extracted spectra across cone voltage acquisitions from the black market pharmaceutical product demonstrating the presence of precursor and in‐source fragmentation‐derived product ions previously identified via (D) liquid chromatography‐tandem mass spectrometry (LC‐MS/MS) analysis.

All samples, including the pharmaceutical products and certified clean supplements, generated detectable ions and presented a typical response of a sharp increase in the total ion chromatogram on glass capillary insertion into the ion source, which returned towards baseline levels over time (Figure 1A,B). Where prohibited substances could be detected via manual interrogation, a clear diagnostic ion pattern was present with the precursor [M+H]^+^ ion present in the 15 V cone voltage channel alongside known product ions present from in‐source fragmentation, present above baseline noise values across channels with increasing cone voltages (see Figure 1C,D for a product containing clomiphene). For the software‐driven identification process, multiple diagnostic assessment screens can be accessed demonstrating the total confidence match against the library entry, as well as the capacity to review the compound matching statistics (total confidence alongside forward and reverse match scores) and sample‐to‐library ion profile comparisons across all data channels (see Figure S1 for examples of a true positive and a true negative identification).

Not one prohibited substance present within the target library was identified in the certified clean nutritional supplements via manual or software‐based interrogation. This demonstrated the capacity for identification workflows to avoid false positives generated by ions present in substance‐clean matrices. A predicted assignment for a target prohibited substance was possible for each pharmaceutical product sample via both the manual and software‐driven approaches. The manual search workflow produced a tentative identification of at least one prohibited substance in each of the pharmaceutical products and was able to correctly assign 68% of substances to their presence, with 56% and 88% correct assignments for samples and AFs, respectively. The use of a software‐based library searching approach significantly improved the ability to correctly identify prohibited substances, with 96%, 69%, and 100% correct identifications for substance, sample and AF, respectively.

An initial investigation into the lack of sample recognition success for the software‐based workflow observed that the [M+H]^+^ ion for trenbolone (*m/z* 271) matched a fragment ion formed from the cleavage of the ester from the testosterone ester compounds. This drove the false positive identification for trenbolone through the (mis)matching of the *m/z* 271 fragment ion from the testosterone esters incorrectly to the protonated trenbolone precursor ion (see Figure S2 for an example false positive output). Given that the trenbolone precursor ion was present in lower cone voltage channels and progressively fragmented with increasing in‐source energy, the library matching criteria was altered to provide a greater emphasis on the reverse match comparison (i.e. the comparison of the library to the unknown sample) to reduce the impact of the variable presence across the data channels of *m/z* 271 dependent on the precursor compound. Initially, the weighting for contribution of the forward:reverse scoring for confidence matching was altered from 70:30 to 60:40; however, this could not resolve the issue for the false positive identification of trenbolone. In addition, ion ratio values calculated for the two most abundant ions in each data channel were included for trenbolone within the searching criteria; however, this was also unable to rectify the false positive identifications. Given the identification that the major mismatching was occurring across acquisition channels where *m/z* 271 was present, the threshold for the reverse match score was increased from 500 to 700 only for trenbolone to increase emphasis on matching for the presence of other ions, reducing the impact of the *m/z* 271 peak resulting in false positive outcomes for trenbolone. This was able to remove all records of false positives for trenbolone identified in the testosterone ester mixes, without altering the success of correct identifications for other substances (see Table S2). This approach was able to improve the correct identification for the sample to 96%. Table 2 provides an overview of the final identifications allocated to each sample for the manual and software‐based response, with Table S3 detailing the diagnostic performance split by individual substances.

**TABLE 2:** Comparison of manual and software‐based workflows for final substance identifications listed for each black market pharmaceutical product. **Bold** indicates a false positive.

Sample ID	Prohibited substances present	Manual substance identification	Software substance identification
1	Testosterone undecanoate	Testosterone undecanoate **Testosterone decanoate**	Testosterone undecanoate
2	Testosterone undecanoate	Testosterone undecanoate **Testosterone decanoate**	Testosterone undecanoate
3	Testosterone undecanoate	Testosterone undecanoate	Testosterone undecanoate
4	Trenbolone	Trenbolone	Trenbolone
5	Testosterone propionate Testosterone phenylpropionate Testosterone decanoate Testosterone isocaproate	Testosterone decanoate Testosterone isocaproate	Testosterone propionate Testosterone phenylpropionate Testosterone decanoate Testosterone isocaproate
6	Testosterone propionate Testosterone phenylpropionate Testosterone decanoate Testosterone isocaproate	Testosterone decanoate Testosterone isocaproate **Trenbolone**	Testosterone propionate Testosterone phenylpropionate Testosterone decanoate Testosterone isocaproate
7	Testosterone propionate Testosterone phenylpropionate Testosterone decanoate Testosterone isocaproate	Testosterone decanoate Testosterone isocaproate	Testosterone phenylpropionate Testosterone decanoate Testosterone isocaproate
8	Ostarine	Ostarine	Ostarine
9	Clenbuterol	Clenbuterol	Clenbuterol
10	Clenbuterol	Clenbuterol	Clenbuterol
11	Clenbuterol	Clenbuterol	Clenbuterol
12	Clenbuterol	Clenbuterol	Clenbuterol
13	Clomiphene	Clomiphene	Clomiphene
14	Clomiphene	Clomiphene	Clomiphene
15	Clomiphene	Clomiphene	Clomiphene
16	Clomiphene	Clomiphene	Clomiphene

Diagnostic performance calculations were performed on the combination of outcomes for identification of prohibited substances in both the pharmaceutical products (i.e. known positives) and the certified clean supplements (i.e. known negatives) (Table 3) using the optimised library search settings. The manual identification reported sensitivity (47%–68%) and PPV (53%–81%) to be low across the three reporting categories for substance, sample, and AF. Specificity (64%–75%) and NPV (47%–70%) values were also low for substance and sample identification, but highlighted that not one certified clean sample was manually assigned a prohibited substance (AF specificity = 100%, NPV = 100%). The software‐driven workflow (including optimisation for false trenbolone identifications) showed excellent performance across all aspects of diagnostic statistics, including flawless identification and exclusion of AFs. The predictive capabilities for the software‐based workflow to identify the correct substance (c‐statistic = 0.980, *p* < 0.01), sample (0.906, *p* < 0.01), and AF (1.000, *p* < 0.01) were all excellent.

**TABLE 3:** Diagnostic performance for the manual and software‐based workflows for the identification of the correct substance (i.e. identification of a substance wherever present), sample (i.e. positive identification of all substances present within a sample), and adverse finding (AF; i.e. positive identification of at least one substance present in a sample).

	Substance		Sample		AF	
	*Manual*	*Software*	*Manual*	*Software*	*Manual*	*Software*
Correct ID	17/25 (68%)	24/25 (96%)	9/16 (56%)	15/16 (94%)	14/16 (88%)	16/16 (100%)
**Sensitivity**	68%	96%	47%	94%	54%	100%
**Specificity**	64%	100%	75%	100%	100%	100%
**PPV**	81%	100%	53%	100%	70%	100%
**NPV**	47%	92%	70%	92%	100%	100%
**C‐statistic**	0.522	0.980^**^	0.635^*^	0.906^**^	0.719*	1.000^**^

Abbreviations: ID = identification; NPV = negative predictive value; PPV = positive predictive value.

* *p* < 0.05.

** *p* < 0.001.

**FIGURE 2:**
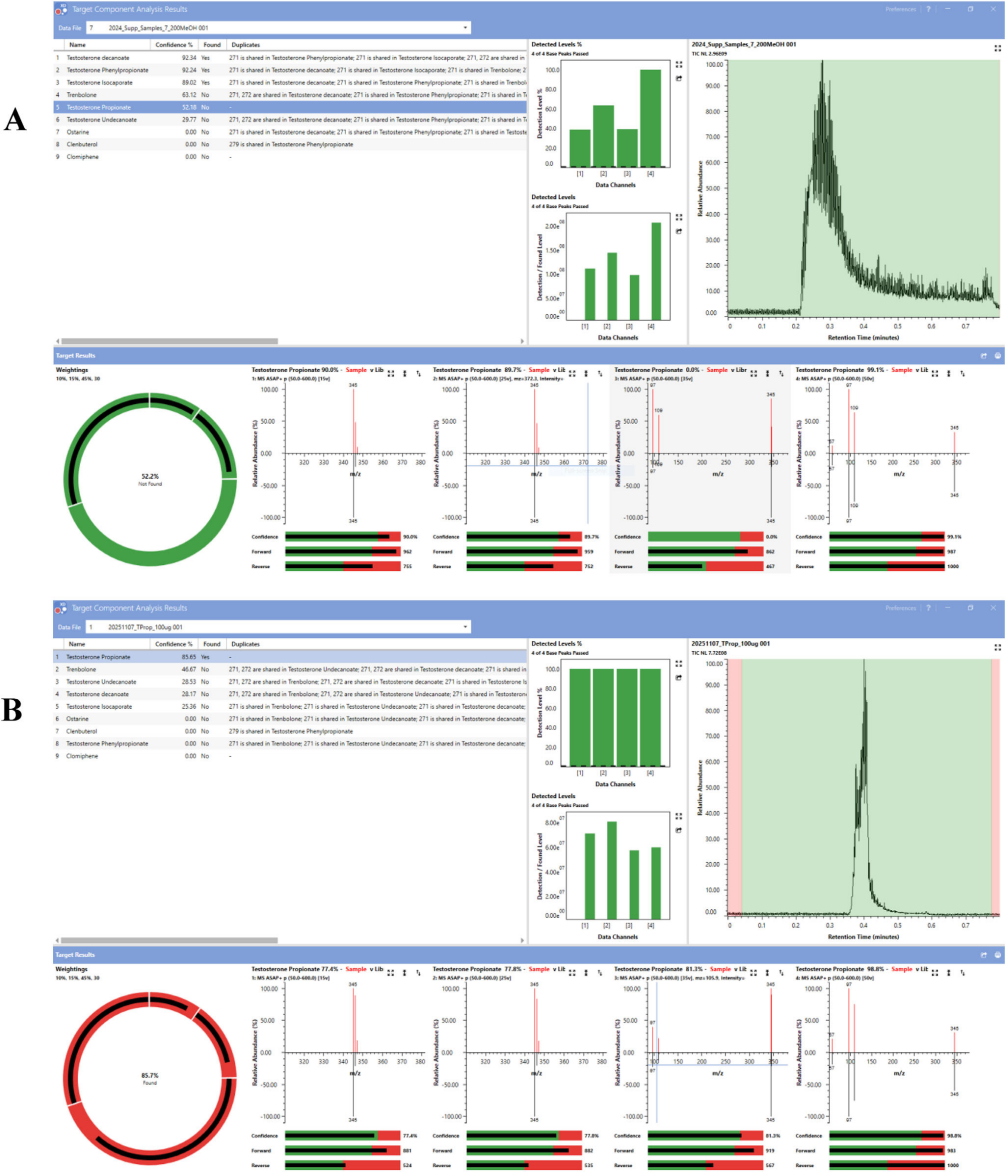
Software‐based library searching output for (A) the false negative outcome for testosterone propionate in a mixed testosterone ester product and (B) the successful capability identification for testosterone propionate via library matching without interference of ions from competing product ions.

**FIGURE 3:**
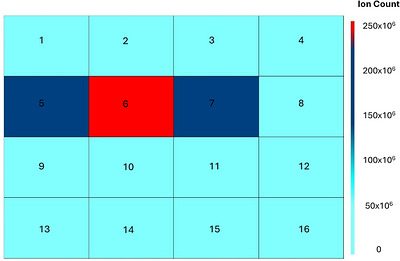
Heatmap generated for the presence of *m/z* 345 in channel 1 (15 V) to support detection of the testosterone propionate [M+H]^+^ diagnostic precursor ion present in mixed testosterone ester pharmaceutical products (samples 5, 6, and 7).

Despite the ability of the software to significantly exceed the manual identification of prohibited substances within the pharmaceutical products, issues with performance characteristics when assessed by each individual prohibited substance were identified. For example, the positive identification for the presence of testosterone propionate demonstrated mixed success (two of three correctly identified), as well as a recurrent issue for the false positive identification of trenbolone in testosterone ester‐containing samples. As samples containing testosterone propionate also contained other testosterone esters at higher dosages, further investigation identified that overlapping of target ion profiles between the prohibited substances sufficiently perturbed ion contributions for *m/z* 97 and *m/z* 109 (Figure 2A). This resulted in the ion statistics for the sample containing testosterone propionate being sufficiently distorted in comparison to the library entry. This meant that whilst an identification for testosterone propionate was flagged, it did not meet the threshold of an 80% confidence match score. Indeed, issues with library matching were ruled out, as when an independent measurement of the testosterone propionate standard was compared to the library entry, a high confidence match score was confirmed (86%, see Figure 2B). A further reporting feature from the software allowed the generation of a heatmap for isolated ions from the data channel of choice. When the precursor ion for testosterone propionate (*m/z* 345) was isolated from the 15 V cone voltage channel, it was possible to clearly identify all samples containing testosterone propionate from a matrix of all samples analysed (see Figure 3). This demonstrated an additional reporting feature to provide support to the library matching workflow, which could be automatically incorporated where a compound match falls close to, but below, a pre‐set match threshold (i.e. tentative identification). However, and importantly in the context of sports anti‐doping, the correct identification of at least one of the prohibited esters remained possible in all mixed testosterone ester products.

## 4 Discussion

This proof‐of‐concept study demonstrated the potential for software‐driven library searching of products analysed by ASAP‐MS to act as a rapid screening tool for the identification of prohibited substances in black market pharmaceutical products. Importantly, the ASAP‐MS approach was able to detect at least one correct prohibited substance within each pharmaceutical product, supporting its applicability for rapid qualitative assessment of seized products in an anti‐doping context. Furthermore, the protocols used required little to no sample preparation, utilised a small footprint instrument capable of deployment outside of the laboratory, and applied a workflow which could be developed to be incorporated into a user‐friendly, non‐expert interface for automated analysis and reporting.

The ability for ASAP‐MS to offer a rapid and reliable identification of drugs has seen increased interest in recent years, notably through the testing of recreational/illicit drugs [11, 16, 20] alongside screening for purity and authenticity of pharmaceutical tablets [14]. The current investigation applied a workflow which investigated pharmaceutical products and was able to provide clear diagnostic ion patterns to identify substances prohibited by WADA, including anabolic agents, and a hormone and metabolic modulator. Importantly, not one of the investigated prohibited substances was identified within the certified‐clean nutritional supplements. This highlighted the ability to exclude erroneous false positives, albeit a truly contaminated supplement may fall below the detection limits of the ASAP‐MS system [21, 22]. Whilst the scope and number of prohibited substances assessed in this project were modest, the excellent capabilities for positive AF identifications highlight the promise for future development of larger compound library databases specific for use within sports drug testing. Indeed, it would be of particular benefit to develop libraries which target specific drugs/drug classes considered at high risk for use across sports, or within specific sports, to ensure clean competition and athlete health.

The instrument and set‐up applied in this work were commercially available and purpose‐built for rapid and simple analyses using the ASAP‐MS approach. Nonetheless, the ability for a trained user (∼2.5 years of experience) to confidently identify diagnostic ion patterns for qualitative confirmation of prohibited substance presence remained limited, with only 68% of substances successfully identified and likely compounded by low target ion intensities at levels within background noise. The application of a software‐based library searching approach was able to far outperform the trained user in terms of correct substance identification, with 96% success rate for the identification of a specific substance present and 100% for identification of an AF, with the latter being of particular importance when considering its potential application in sports anti‐doping workflows (i.e. presence of *any* prohibited substance is sufficient for sanction). Importantly, manual searching involved a relatively time‐consuming process, with the time taken to process the samples via library searching considerably less burdensome. For specific application in sports drug testing, particularly related to in‐competition testing where a large number of samples analysed per day would be advantageous, the rapid library search‐and‐response (∼2 min), alongside minimal sample preparation, offers real potential for the implementation of high‐throughput ASAP‐MS analyses into anti‐doping workflows that can be deployed outside of the laboratory setting [23]. However, additional developments to improve analytical sensitivity, especially for samples within complex matrices, are required before this approach can be applied to athlete biological samples where drugs will be present at very low levels.

Whilst this is not the first application of a small form factor ASAP‐MS in drug identification [11, 13, 16, 24, 25], previous studies have used a proprietary software (Waters LiveID) which performs spectral matching but does not allow extensive tailoring of search algorithm functions to improve performance; whereas, the current investigation utilised a third‐party software programme (SpectralWorks AnalyzerPro XD) which allowed such modifications to library matching criteria. This proved important for the current application due to the recurrent false positive identification of trenbolone where other testosterone‐based drugs were present. The alteration of the reverse match threshold value for trenbolone allowed this issue to be negated, without the loss of trenbolone identification when present. Indeed, the individual substance optimisation was required as a blanket increase in reverse match scores across all prohibited substances resulted in reduced capability for true positive identifications (data not shown). In addition, the generation of heatmaps for a known diagnostic ion further improved the capability of the current approach to detect the presence of a prohibited substance. For example, it was not possible to identify the presence of testosterone propionate in one mixed testosterone ester product due to interfering fragment ion profiles from similarly structured molecules. A heatmap generated for *m/z* 345 (protonated precursor ion for testosterone propionate) in channel 1 clearly identified the three samples which contained testosterone propionate, providing excellent utility as a secondary screening approach in addition to library matching. Further development of the screening process could allow the application of a user‐friendly graphical user interface, which automatically screens submitted samples within a known library, including both library matching and diagnostic ion heatmaps within a default reporting function to allow easy access/use for non‐specialist operators.

Heatmaps have been previously used with chromatography‐coupled mass spectrometry data to assess impurities within seized drugs [26]. For ambient techniques, this feature has been used with DART‐ToF MS alongside a spectral library search to visually inspect for the presence of source oils within unknown wood samples [27]. Whilst these studies utilised the rapid nature of visual inspection, the heatmaps were both displayed in a format displaying a range of *m/z* values across multiple samples in one plot. In this work, the heatmap visualisation feature is further simplified by visualising the intensity of a single *m/z* value from a diagnostic ion, as opposed to a range of values. This interface could benefit users by the simplistic format of specific ion searching and could be integrated into a standard report feature alongside the library result to offer an additional user‐friendly format to the screening process. However, and importantly, the inability to identify the one case of testosterone propionate did not compromise the identification of an AF, with successful identification of the other testosterone esters present within the product. From an anti‐doping perspective, the primary utility of a high‐throughput screening tool (especially when deployed outside of the laboratory) is to flag suspect samples/products for further confirmatory analyses, rather than definitive substance(s) identification. Therefore, the ability for ASAP‐MS to reliably identify the presence of any one testosterone ester within these products is arguably of greater operational relevance than perfect discrimination between structurally related drugs; indeed, additional reporting functions to flag suspected drug classes (e.g. a testosterone‐based drug) would provide further utility for screening purposes.

## 5 Conclusions

This proof‐of‐concept study demonstrated the utility of ASAP‐MS as a promising complementary approach to existing anti‐doping workflows, particularly for the consideration of rapidly identifying the potential presence of prohibited substances within products seized from athletes/support personnel. The workflow implemented straightforward sample preparation steps and short analysis times, coupled with fast and reliable identification of prohibited substances. Whilst the technique is not considered to act as a replacement for laboratory‐based confirmatory analyses, it offers clear advantages in speed, deployability, and potential for use by non‐specialist personnel. Future developments should focus on expanding compound libraries, including the development of specific libraries to target substances known to be currently at high risk of use in sport, as well as testing their utility and performance outside of the laboratory setting. Overall, rapid screening of prohibited substances using ASAP‐MS shows exciting promise for implementation into anti‐doping protocols as a complementary high‐throughput product/sample screening approach.

## Author Contributions

**Mario Thevis**: conceptualisation, supervision, project administration, resources, and writing – review and editing. **Oliver Krug**: data curation, project administration, resources, and writing – review and editing. **Ashley Sage**: methodology, resources, and writing – review and editing. **John Moncur**: methodology, software, resources, and writing – review and editing. **David Douce**: methodology, resources, and writing – review and editing. **Scott J. Campbell**: methodology, software, resources, writing review and editing. **Alisha Henderson**: methodology, data curation, investigation, formal analysis, and writing – original draft. **Liam M. Heaney**: conceptualization, methodology, formal analysis, supervision, project administration, resources, writing – original draft, writing – review and editing.

## Ethics Statement

The work described in this manuscript did not employ the use of human or animal participants.

## Conflicts of Interest

The authors declare no conflicts of interest.

## Supporting information




**Supporting File**: ansa70088‐sup‐0001‐SuppMat.docx.

## Data Availability

The data that support the findings of this study are available from the corresponding author upon reasonable request.

## References

[1] O. de Hon , H. Kuipers , and M. van Bottenburg , “Prevalence of Doping Use in Elite Sports: A Review of Numbers and Methods,” Sports Medicine 45, no. 1 (2015): 57–69, 10.1007/s40279-014-0247-x. 25169441

[2] F. Lauritzen and A. Solheim , “The Purpose and Effectiveness of Doping Testing in Sport,” Frontiers in Sports and Active Living 6 (2024): 1386539, 10.3389/fspor.2024.1386539. 38803418 PMC11128570

[3] World Anti‐Doping Agency . 2023 Anti‐Doping Testing Figures. 2025, accessed February 10, 2026, https://www.wada‐ama.org/sites/default/files/2025‐06/2023_anti_doping_testing_figures_en_0.pdf.

[4] L. B. Patterson and S. H. Backhouse , “An Important Cog in the Wheel‘, but Not the Driver: Coaches’ perceptions of Their Role in Doping Prevention,” Psychology of Sports and Exercise 37 (2018): 117–127, 10.1016/j.psychsport.2018.05.004.

[5] D. A. Baron and D. M. Martin , “Abol Magd S. Doping in Sports and Its Spread to at‐risk Populations: An International Review,” World Psychiatry 6, no. 2 (2007): 118–123. 18235871 PMC2219897

[6] World Anti‐Doping Agency , Performance‐Enhancing Drug Trafficking on the Dark Web—Public Report. 2022, accessed June 26, 2025, https://www.wada‐ama.org/en/resources/performance‐enhancing‐drug‐trafficking‐dark‐web‐public‐report#resource‐download.

[7] A. B. M. Merlo , L. Lobigs , T. Piper , C. Champod , and N. Robinson , “Unravelling the Threat of Contamination in Elite Sports: Exploring Diverse Sources Impacting Adverse Analytical Findings and the Risk of Inadvertent Exposure to Prohibited Substances,” Forensic Science International 365 (2024): 112240, 10.1016/j.forsciint.2024.112240. 39442273

[8] E. Petkova‐Gueorguieva , S. Gueorguiev , H. Lebanova , A. Mihaylova , and V. Madzharov , “Investigation of the Content of Anabolic Steroids in Food Supplements Used in Sports Practice,” All Life 16, no. 1 (2023): 2270722, 10.1080/26895293.2023.2270722.

[9] C. L. Torres , F. A. G. de Oliveira , L. F. Jooris , M. C. Padilha , and H. M. G. Pereira , “The Presence of Doping Agents in Dietary Supplements: A Glimpse Into the Brazilian Situation,” Drug Testing and Analysis 16, no. 1 (2024): 38–48, 10.1002/dta.3517. 37161689

[10] W. Schänzer and M. Thevis , “Human Sports Drug Testing by Mass Spectrometry,” Mass Spectrometry Reviews 36, no. 1 (2017): 16–46, 10.1002/mas.21479. 26213263

[11] A. Frinculescu , B. Mercer , T. Shine , et al., “Assessment of a Single Quadrupole Mass Spectrometer Combined With an Atmospheric Solids Analysis Probe for the on‐Site Identification of Amnesty Bin Drugs,” Journal of the American Society for Mass Spectrometry 35, no. 7 (2024): 1480–1489, 10.1021/jasms.4c00064. 38837752 PMC11228975

[12] B. J. McCullough , K. Patel , R. Francis , et al., “Atmospheric Solids Analysis Probe Coupled to a Portable Mass Spectrometer for Rapid Identification of Bulk Drug Seizures,” Journal of the American Society for Mass Spectrometry 31, no. 2 (2020): 386–393, 10.1021/jasms.9b00020. 32031401

[13] F. Smillie , W. Glinka , C. Henry , A. McCudden , J. Thorpe , and S. W. Holman , “Demonstration of an End‐To‐End Workflow Using Atmospheric Solids Analysis Probe–Mass Spectrometry (ASAP‐MS) with Real‐Time Sample Recognition Software for the Identification of Falsified and Substandard Pharmaceutical Tablets,” Drug Testing and Analysis 17, no. 7 (2025): 1096–1106, 10.1002/dta.3816. 39394933

[14] S. Mathias , D. Burns , T. Hambidge , et al., “Assessment of Atmospheric Solids Analysis Probe as a Tool for the Rapid Determination of Drug Purity,” Drug Testing and Analysis 16, no. 8 (2024): 807–816, 10.1002/dta.3568. 37621075

[15] D. Burns , S. Mathias , B. J. McCullough , et al., “Ambient Ionisation Mass Spectrometry for the Trace Detection of Explosives Using a Portable Mass Spectrometer,” International Journal of Mass Spectrometry 471 (2021): 116735, 10.1016/j.ijms.2021.116735.

[16] R. Koerber and G. F. Verbeck , “Fentanyl and Fentanyl Analogue Screening Using ASAP‐MS with LiveID Confirmation,” Rapid Communications in Mass Spectrometry 39, no. 9 (2025): e9994, 10.1002/rcm.9994. 39873905

[17] A. Henderson , L. M. Heaney , and S. Rankin‐Turner , “Ambient Ionisation Mass Spectrometry for Drug and Toxin Analysis: A Review of the Recent Literature,” Drug Testing and Analysis 16, no. 11 (2024): 1323–1344, 10.1002/dta.3644. 38326879

[18] A. Henderson , L. M. Heaney , and S. Rankin‐Turner , “Advancements in Ambient Ionisation Mass Spectrometry in 2024: An Annual Review,” Analytical Science Advances 6, no. 1 (2025): e70007, 10.1002/ansa.70007. 40123829 PMC11929545

[19] C. Weber , O. Krug , M. Kamber , and M. Thevis , “Qualitative and Semiquantitative Analysis of Doping Products Seized at the Swiss Border,” Substance Use & Misuse 52, no. 6 (2017): 742–753, 10.1080/10826084.2016.1263665. 28156209

[20] R. D. Soares , D. K. John , M. P. Thomé , P. S. Corrêa , K. S. Souza , and M. F. Ferrão , “Robust Detection of Ecstasy‐Like and Adulterants through ASAP‐MS and DD‐SIMCA,” Drug Testing and Analysis 17, no. 9 (2025): 1567–1574, 10.1002/dta.3860. 39905800

[21] H. Geyer , M. K. Parr , K. Koehler , U. Mareck , W. Schänzer , and M. Thevis , “Nutritional Supplements Cross‐contaminated and Faked With Doping Substances,” Journal of Mass Spectrometry 43, no. 7 (2008): 892–902, 10.1002/jms.1452. 18563865

[22] A. Henderson , S. Rankin‐Turner , J. C. Reynolds , et al., “Assessing the Quantitative Performance of Atmospheric Solids Analysis Probe‐Mass Spectrometry,” Rapid Communications in Mass Spectrometry 39, no. 22 (2025): e10112, 10.1002/rcm.10112. 40746218 PMC12314589

[23] S. Rankin‐Turner and L. M. Heaney , “Deployable Mass Spectrometry for Rapid on‐Site Bioanalysis,” LCGC North America 40 (2022): 14–18, 10.56530/lcgc.na.lt8569n1.

[24] X. Wang , M. Huang , X. Li , W. Dai , and J. Liang , “Rapid Screening of Illegal Additives in Health Products Using Atmospheric Pressure Solids Analysis Probe Coupled to a Portable Mass Spectrometer,” SSRN Journal 214 (2022): 1–35, 10.2139/ssrn.4014203. 35325799

[25] A. Arrizabalaga‐Larrañaga , P. W. Zoontjes , J. J. P. Lasaroms , M. W. F. Nielen , and M. H. Blokland , “Simplified Screening Approach of Anabolic Steroid Esters Using a Compact Atmospheric Solid Analysis Probe Mass Spectrometric System,” Analytical and Bioanalytical Chemistry 414, no. 11 (2022): 3459–3470, 10.1007/s00216-022-03967-y. 35220465 PMC9018663

[26] T. Y. Huang and J. C. C. Yu , “Intelligent Framework for Cannabis Classification Using Visualization of Gas Chromatography/Mass Spectrometry Data and Transfer Learning,” Frontiers in Analytical Science 3 (2023): 1125049, 10.3389/frans.2023.1125049.

[27] E. R. Price , P. J. McClure , A. N. Huffman , D. Voin , and E. O. Espinoza , “Reliability of Wood Identification Using DART‐TOFMS and the ForeST© Database: A Validation Study,” Forensic Science International: Animals and Environments 2 (2022): 100045, 10.1016/j.fsiae.2022.100045.

