# Supplementary material for: Rapid Analysis of Sports Prohibited Substances in Black Market Pharmaceutical Products Using Atmospheric Solids Analysis Probe‐Mass Spectrometry
Source: Anal Sci Adv. 2026 Apr 28;7(1):e70088. doi: 10.1002/ansa.70088 (PMC13124081; doi:10.1002/ansa.70088)
Supplement: Supplementary file 1 — Supporting File: ansa70088‐sup‐0001‐SuppMat.docx. [file ANSA-7-e70088-s001.docx]

**SUPPLEMENTARY INFORMATION**

**Rapid Analysis of sports prohibited substances in black market pharmaceutical products using ASAP-MS**

Alisha Henderson, Oliver Krug, Ashley Sage, David Douce, Scott J Campbell, John Moncur, Mario Thevis, Liam M Heaney

**Table S1**. List of analysed prohibited substances and respective mass spectrometric properties. All structures were drawn according to SDF co-ordinates obtained from PubChem and drawn in ChemSketch (v2024.1.4, ACD/Labs, Toronto, ON, Canada).

| **Prohibited Substance** | **Chemical Structure** | **Monoisotopic Mass (Da)** | **[M+H]^+^ Precursor Ion** | **Product Ions** |
| --- | --- | --- | --- | --- |
| Clenbuterol (S1) |  | 276.08 | *m/z* 277.1 | *m/z* 132.1  *m/z* 168.0  *m/z* 203.0 |
| Ostarine (S1) |  | 389.10 | *m/z* 390.1 | *m/z* 118.0  *m/z* 185.0  *m/z* 269.1 |
| Trenbolone (S1) |  | 270.16 | *m/z* 271.16 | *m/z* 159.1  *m/z* 199.1  *m/z* 253.2 |
| Testosterone propionate (S1) |  | 344.24 | *m/z* 345.24 | *m/z* 57.0  *m/z* 97.1  *m/z* 109.1  *m/z* 271.2 |
| Testosterone phenylpropionate (S1) |  | 420.27 | *m/z* 421.27 | *m/z* 97.1  *m/z* 105.1  *m/z* 109.1  *m/z* 271.2 |
| Testosterone decanoate (S1) |  | 442.34 | *m/z* 443.34 | *m/z* 71.1  *m/z* 97.1  *m/z* 109.1 |
| Testosterone undecanoate (S1) |  | 456.36 | *m/z* 457.36 | *m/z* 97.1  *m/z* 109.1  *m/z* 175.1 |
| Testosterone isocaproate (S1) |  | 386.28 | *m/z* 387.28 | *m/z* 97.1  *m/z* 81.1  *m/z* 109.1 |
| Clomiphene (S4) |  | 405.19 | *m/z* 406.19 | *m/z* 58.1  *m/z* 86.1  *m/z* 100.1 |


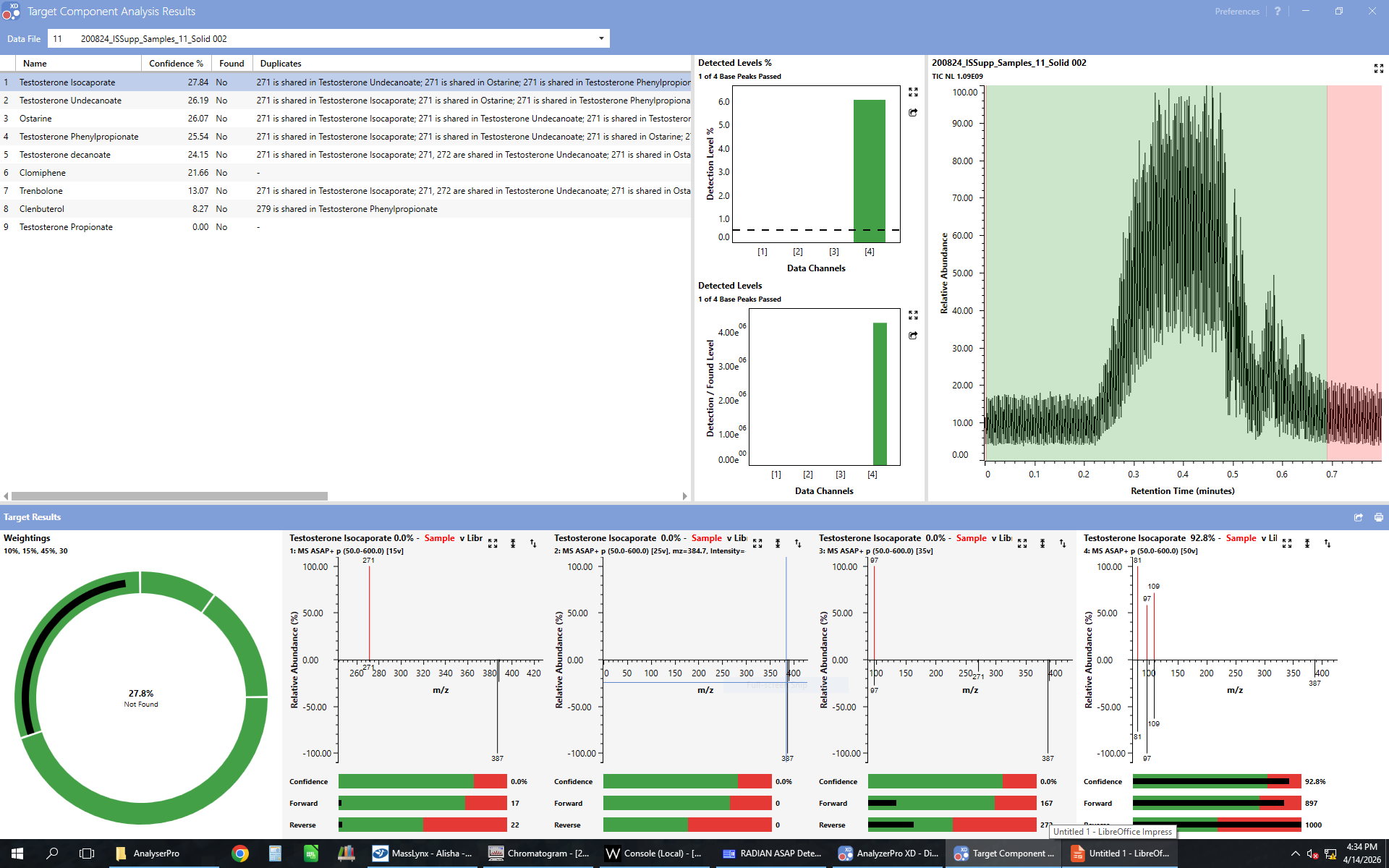


**
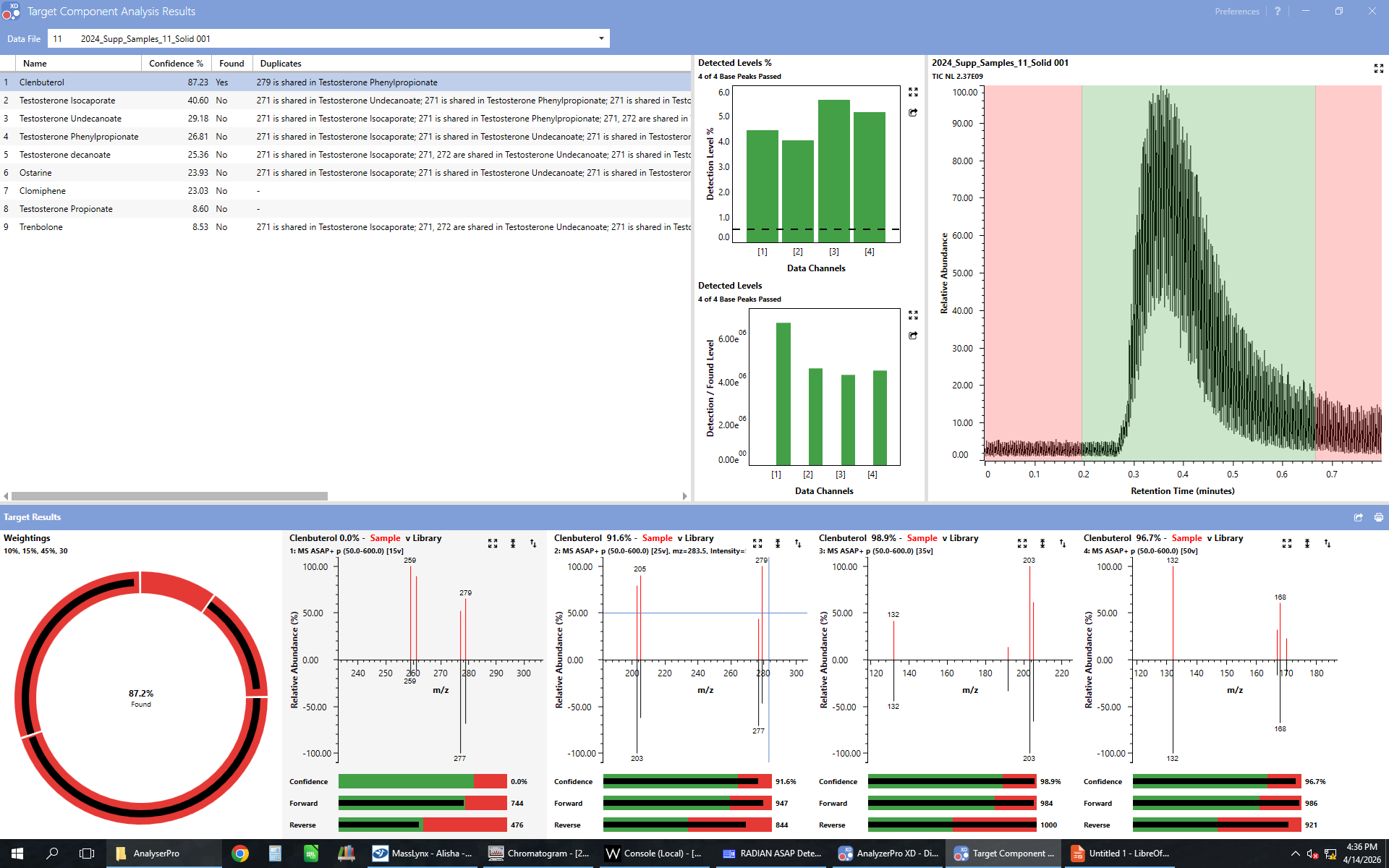
Figure S1.** Example software-based searching outputs to show (**Top**) a true negative result from a certified-clean nutritional supplement and (**Bottom**) a true positive result from a black market pharmaceutical product.


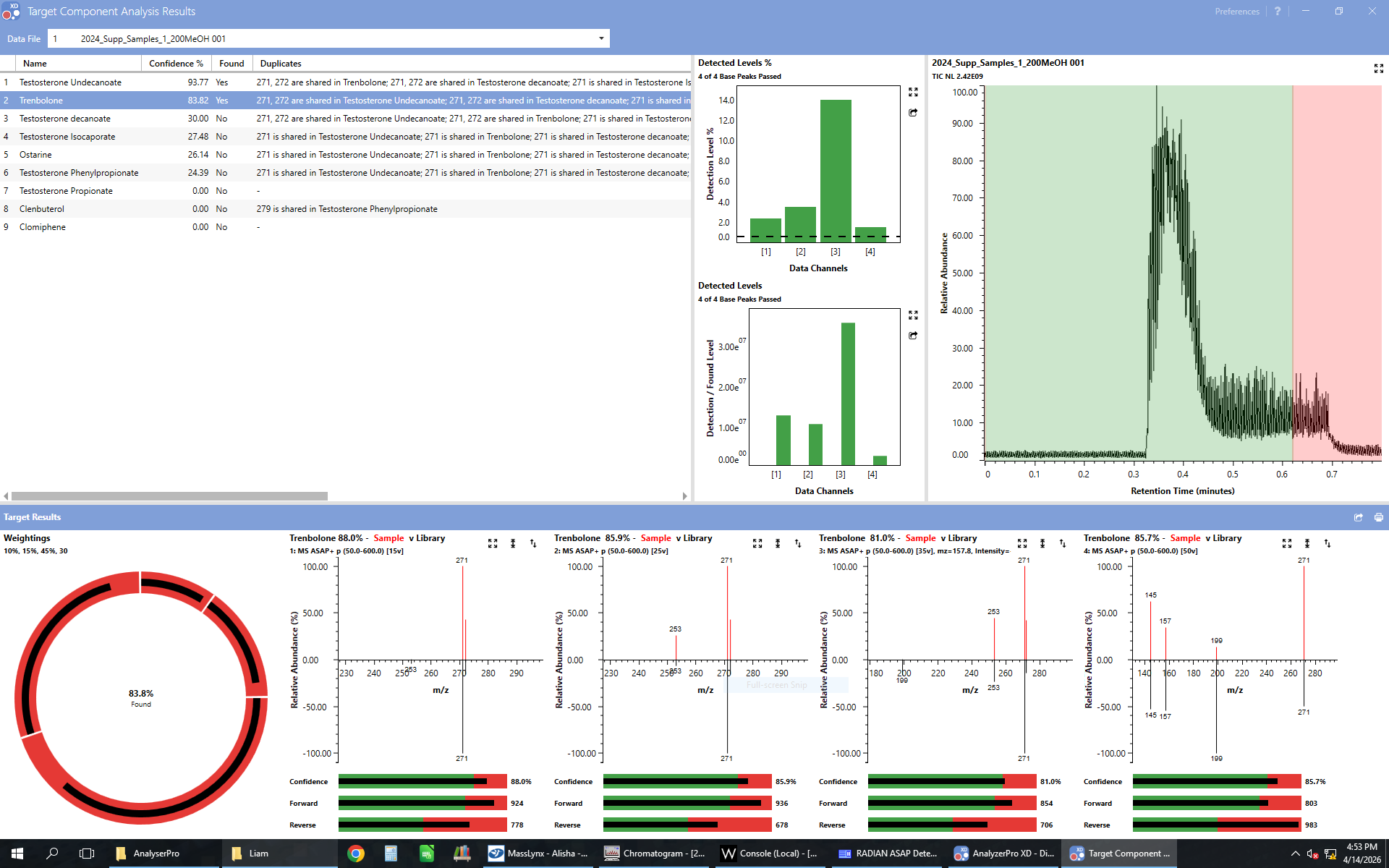
**Figure S2.** Example software-based searching outputs to show a false positive result from a black market pharmaceutical product.

**Table S2**. Summary of identifications of prohibited substances in black market pharmaceutical products using the software-driven approach for the default and optimised library searching functions. The match score value refers to the confidence score reported by the software. Bold text identifies a **false positive (FP)** result and italicised text identifies a *false negative (FN)* result.

| **Sample ID** | **Prohibited Substances Present** | **Default Library Search** | | **Optimised Library Search** | |  |
| --- | --- | --- | --- | --- | --- | --- |
|  |  | *Predicted Content* | *Match Score* | *Predicted Content* | *Match Score* |  |
| 1 | Testosterone undecanoate | Testosterone undecanoate  **Trenbolone (FP)** | 93.8%  **83.8%** | Testosterone undecanoate | 93.8% |  |
| 2 | Testosterone undecanoate | Testosterone undecanoate  **Trenbolone (FP)** | 93.3%  **82.5%** | Testosterone undecanoate | 93.3% |  |
| 3 | Testosterone undecanoate | Testosterone undecanoate  **Trenbolone (FP)** | 93.6%  **82.7%** | Testosterone undecanoate | 93.6% |  |
| 4 | Trenbolone | Trenbolone | 83.2% | Trenbolone | 83.2% |  |
| 5 | Testosterone propionate  Testosterone phenylpropionate  Testosterone decanoate  Testosterone isocaproate | Testosterone propionate Testosterone phenylpropionate  Testosterone decanoate  Testosterone isocaproate  **Trenbolone (FP)** | 91.3%  83.3%  91.2%  87.0%  **83.8%** | Testosterone propionate Testosterone phenylpropionate  Testosterone decanoate  Testosterone isocaproate | 83.3%  91.6%  91.3%  87.0% |  |
| 6 | Testosterone propionate  Testosterone phenylpropionate  Testosterone decanoate  Testosterone isocaproate | Testosterone propionate  Testosterone phenylpropionate  Testosterone decanoate  Testosterone isocaproate | 88.1%  90.8%  89.8%  86.8% | Testosterone propionate  Testosterone phenylpropionate  Testosterone decanoate  Testosterone isocaproate | 88.1%  90.8%  89.8%  86.8% | |
| 7 | Testosterone propionate  Testosterone phenylpropionate  Testosterone decanoate  Testosterone isocaproate | *Testosterone propionate (FN***)**  Testosterone phenylpropionate  Testosterone decanoate  Testosterone isocaproate  **Trenbolone (FP)** | *52.2%*  92.3%  92.3%  89.0%  **83.8%** | *Testosterone propionate (FN***)**  Testosterone phenylpropionate  Testosterone decanoate  Testosterone isocaproate | *52.2%*  92.3%  92.3%  89.0% | |
| 8 | Ostarine | Ostarine | 92.6% | Ostarine | 92.6% | |
| 9 | Clenbuterol | Clenbuterol | 97.0% | Clenbuterol | 97.0% | |
| 10 | Clenbuterol | Clenbuterol | 95.1% | Clenbuterol | 95.1% | |
| 11 | Clenbuterol | Clenbuterol | 87.2% | Clenbuterol | 87.2% | |
| 12 | Clenbuterol | Clenbuterol | 81.3% | Clenbuterol | 81.3% | |
| 13 | Clomiphene | Clomiphene | 97.0% | Clomiphene | 97.0% | |
| 14 | Clomiphene | Clomiphene | 97.2% | Clomiphene | 97.2% | |
| 15 | Clomiphene | Clomiphene | 95.8% | Clomiphene | 95.8% | |
| 16 | Clomiphene | Clomiphene | 97.3% | Clomiphene | 97.3% | |

**Table S3**. Summary of diagnostic performance statistics for the identification of individual prohibited substances of interest in black market pharmaceutical products using the optimised and software-driven library matching approach.

|  | **Prohibited Substance** | | | | | | | | |
| --- | --- | --- | --- | --- | --- | --- | --- | --- | --- |
|  | *Trenbolone* | *T propionate* | *T phenylpropionate* | *T decanoate* | *T undecanoate* | *T isocaproate* | *Ostarine* | *Clenbuterol* | *Clomiphene* |
| **Specificity** | 100% | 100% | 100% | 100% | 100% | 100% | 100% | 100% | 100% |
| **Sensitivity** | 100% | 67% | 100% | 100% | 100% | 100% | 100% | 100% | 100% |
| **PPV** | 100% | 100% | 100% | 100% | 100% | 100% | 100% | 100% | 100% |
| **NPV** | 100% | 96% | 100% | 100% | 100% | 100% | 100% | 100% | 100% |
| **Efficiency** | 100% | 96% | 100% | 100% | 100% | 100% | 100% | 100% | 100% |

Note: NPV = negative predictive value; PPV = positive predictive value
